# Letter from a Patient to his Physician

**Published:** 1808-04

**Authors:** 


*The following Letter from a Patient to his Physician will probably amuse some of your Readers and inform others.*

*March* 3, 1808.

To Dr. ———

My dear Sir,

Last night I accidentally met with the following passage in a book, the leaves of which I was carelessly turning over: "If a physician neglecteth regularly to receive his fees, and his patient recovereth, he hath no right to sue sue him afterwards; but if the patient *dieth*, the physician hath a legal claim to remuneration." It would be difficult to express the alarm this excited in my breast, especially when what I had so lately read in the Censorian Letter came fresh in my recollection, that physicians, unlike other professional men, carry on their operations in *secret*, and even a man of Dr. Gregory's reputation hints at forming a partnership with an *Undertaker*. All my confidence in your honour and integrity could not, I confess, get the better of my apprehensions; I past a sleepless night, and now hasten to beg your acceptance of the enclosed.* It is, indeed, but an inadequate return for all your attention, and that of your worthy friend, to us for some time past: but I am in hopes it may induce you to spare me a little longer in the land of the living; for I must own, it would grievously vex me to be sent out of the world by an unaccountable non-descript disorder like the present, which I had not even the merit of bringing on myself.

Believe me always with much esteem,

My dear Sir, &c.

——— ———.

* Eighteen guineas.

